# Trends and strategies in the effluent treatment of pulp and paper industries: A review highlighting reactor options

**DOI:** 10.1016/j.crmicr.2021.100077

**Published:** 2021-10-23

**Authors:** Kartik Patel, Niky Patel, Nilam Vaghamshi, Kamlesh Shah, Srinivas Murthy Dugdirala, Pravin Dudhagara

**Affiliations:** a Department of Biosciences (UGC-SAP-II & DST-FIST-I), Veer Narmard South Gujarat University, Surat 395007, India; b P.S. Science and H.D. Patel Arts College Kadi, Mahesana, Gujarat, India; c Biogas Research Centre, Post-Graduate Department of Microbiology, M.D. Gramsewa Mahavidyalaya, Gujarat Vidyapith Sadra, Gandhinagar, Gujarat, India

**Keywords:** Pulp and paper industry, Wastewater, Microbial technology, Membrane bioreactor, Moving-bed biofilm reactor

## Abstract

From the beginning of the paper-making process, the pulp and paper industry has utilized a large amount of water and generated a vast amount of highly polluted wastewater. The paper industry faces global pressure to reduce water use and lower environmental pollution. However, traditional physicochemical methods of wastewater treatment need high energy input, and their ecological impact is questionable. Due to the zero discharged policy, the industries urgently require novel eco-friendly, sustainable, and efficient treatment techniques. Microbial technology is the most recommended option to treat wastewater and support sustainable growth. The present article describes the overview of traditional and novel methods, including membrane bioreactor (MBR) and moving-bed biofilm reactor (MBBR) technology's with their current state and their limits for treating pulp and paper wastewater. It is expected to integrate the novel methods with advanced hybrid technology to fulfill wastewater treatment criteria and prospects. Furthermore, coupling MBR and MBBR technology make energy and water recovery possible, and recycling wastewater will be economically and environmentally feasible.

## Highlights

• Pulp and paper mills generate huge amount of wastewater.
• Physicochemical processes have several limitations to treat wastewater.
• Integrated microbial based technology improves the quality of wastewater.
• Eco-friendly MBBR process creates opportunity for reuse of treated wastewater.

## Graphical abstract

**Figure:**
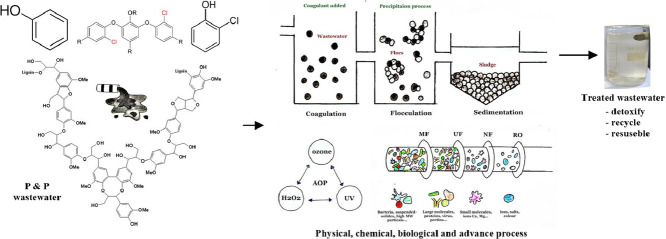

## Introduction

In the last several decades, extensive industrial development has taken place at a rapid speed worldwide. This industrial revolution has disturbed the environment from earlier conditions. Globally, the pulp and paper industry is well established and considered as one of the most important sectors (Gupta and Gupta, 2019). Environmental pollution is one of the significant concerns associated with this industry with freshwater utilization (Negi and Suthar, 2018). The pulp and paper industry typically required a vast amount of water during various operational stages such as washing, pulping, bleaching, and paper-making. According to available data, the pulp and paper industry consumes 250–300 m^3^ of water to produce 1 ton of paper (Chaudhry and Paliwal, 2018). As a result, substantial liquid waste is discharged into the environment, containing numerous harmful chemicals. According to the Ministry of Environmental and Forest, India (MoEF), the pulp and paper industries have come under the "Red Category" in the directory of 17 most polluted industries based on toxic emissions. Literature showed that more than 250 chemical compounds are generated at different stages of the paper-making process (Kumar et al., 2018). Xenobiotic compounds such as chlorinated lignin, chlorinated phenol, chlorinated resin acid, dioxins, chlorophenols, phenols, adsorbable organic halogens (AOX), and extractable organic acids, halogens, metal ions, etc. is generated with lignin and other naturally occurring polymers (Chaudhry and Paliwal, 2018). These compounds are released in water bodies due to the industry's inappropriate or absence of a wastewater treatment system. As a result, the wastewater is exceptionally contaminated with toxic compounds and lignin, which have high BOD and COD contents. The dark brown colored effluent harms aquatic life by restricting photosynthesis, changes the water pH and decreasing the dissolved oxygen level (Haq and Raj, 2020). However, in the developed countries in Europe and North America have well-established wastewater treatment facilities and also there environmental control authorities set strict restrictions on the discharges of chlorinated compounds (Cabrera, 2017).

Therefore, it's necessary to require proper treatment of wastewater to minimize environmental damage. Consequently, it is essential to remove; otherwise, it may lead to a severe threat to the environment, such as loss of environmental aesthetics, adverse effect on aquatic life, increase soil salinity by nutrients imbalance which ultimately affects on economic wealth of a country (Mahesh et al., 2016; Patel and Dudhagara, 2020). Answering these concerns, the environmental researcher is constantly working on various mechanisms to treat or recycle pulp and paper wastewater. Among the possible treatments, microbial groups fungi and bacteria are considered a significant prospect due to their biotransformation and biodegradation efficiency (Mir-Tutusaus et al., 2018). Low-cost reactors and sequencing processes using biological treatment are worth utilizing at an industrial scale.

This study reviews the in-depth knowledge about the various existing strategies to treat pulp and paper industry wastewater and focuses on eco-friendly "bio-based" treatment with a successful application example.

## Waste generation

**Fig. 1:**
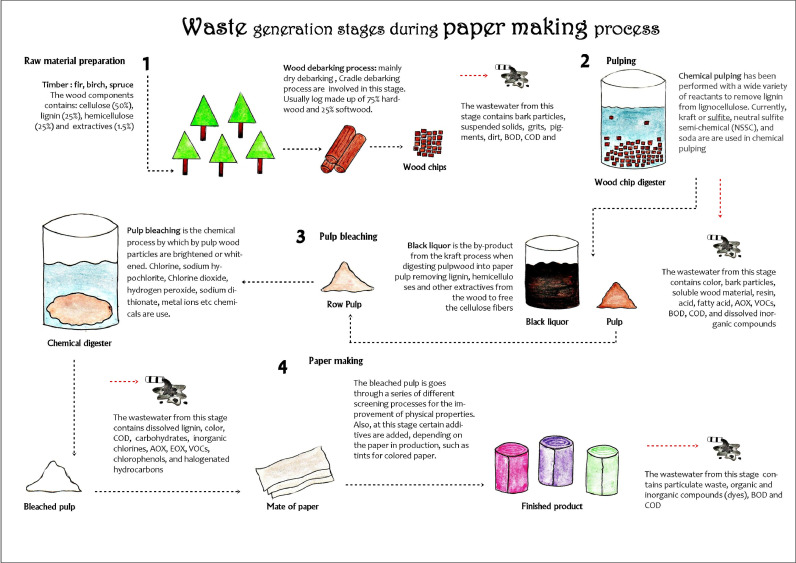
Wastewater generation in various stages of pulp and paper manufacturing process.

The pulp and paper industry generates various waste during the paper-making process at different operational stages, as illustrated in Fig. 1. A considerable volume of wastewater is generated in wood chip preparation, pulping, bleaching, and paper-making steps. The characteristics of discharged wastewater from pulp and paper industries reported by researchers are summarized in Table 1. During the wood chip preparation, washing, and pulping process, lignin and hemicelluloses were separated from the wood chips by NaOH or Na_2_S treatment under alkaline conditions (karft process). At this stage, generated lignin-rich effluent known as black liquor. In the bleaching process, the pulp is treated with hazardous chemicals such as chlorine, hydrogen peroxide, ozone, calcium oxide, hydrochloric acid, etc. (Virkutyte, 2017), adding more toxicity to the final collected effluents. However, the properties of generated wastewater from different process stages depend on the type of raw material, pulping process, the recirculation of effluent and the amount of water used (Pokhrel and Viraraghavan, 2004; Hubbe et al., 2016). The wastewater contains high values of chlorine compounds, BOD, and COD that are accumulatively called as AOX, which generally corresponding to the chlorine consumption in bleaching process (Chaudhry and Paliwal, 2018). The COD value is an important for any wastewater treatment which represents the numbers of organic pollutants present in a wastewater. The ratio of BOD to COD is an index of the presentation of biodegradation which refers as “biodegradability index” (Patel and Patel, 2020). These biodegradability index represent the fraction of organic compounds in the wastewater that are easy to degrade. According to the Dahlman et al. (1995) report, chemical pulping processes generate more than 40% of poorly biodegradable organic compounds within the total organic matter of the wastewater.

**Table 1:** Typical characteristics of wastewater effluents from pulp and paper industries.

Refs.	pH	color (co-pt)	BOD (mg L^−1^)	COD (mg L^−1^)	Lignin (mg L^−1^)	Phenol (mg L^−1^)	Sulfate (mg L^−1^)	Total solids (mg L^−1^)
Eskelinen et al. (2010)	6.4–7.3	660–1230	142–221	1170–1510	133–265	-	-	354–563
Chandra and Singh (2012)	7 ± 0.20	8942 ± 15.0	8296 ± 45	22,189 ± 39	1124 ± 12.09	364 ± 20.13	1089 ± 19.67	1799 ± 17.61
Garg et al. (2012)	7.4 ± 0.1	1761 ± 2.3	185 ± 2.9	2420 ± 17	-	-	-	2359 ± 14.2
Raj et al. (2014)	8.2 ± 1.0	2242 ± 56	385 ± 12	792 ± 70	436 ± 18	42 ± 2.5	993 ± 6	850 ± 30
Yadav and Chandra (2015)	8.5 ± 1.0	2538 ± 53.3	7250 ± 123.0	16,550 ± 507.2	800 ± 18.4	-	1003 ± 5.3	977 ± 7.2
Rajwar et al. (2017)	9.8 ± 1.3	7253 ± 64.2	2934 ± 38.3	6735 ± 45.3	1863 ± 52.6	-	852.4 ± 34.1	1753 ± 34.2
Kumar et al. (2018)	7.7 ± 0.02	1202 ± 6.53	180.54 ± 4.70	584 ± 3.62	-	-	-	1686 ± 10.58
Zalnith et al. (2019)	8.1 ± 1.0	1065 ± 89.3	426 ± 30.6	774 ± 43.7	529 ± 20.10	39.3 ± 12.0	866 ± 47.0	859 ± 45.3

## Methods use in wastewater treatments

### Physical, chemical, and electrochemical process

**Fig. 2:**
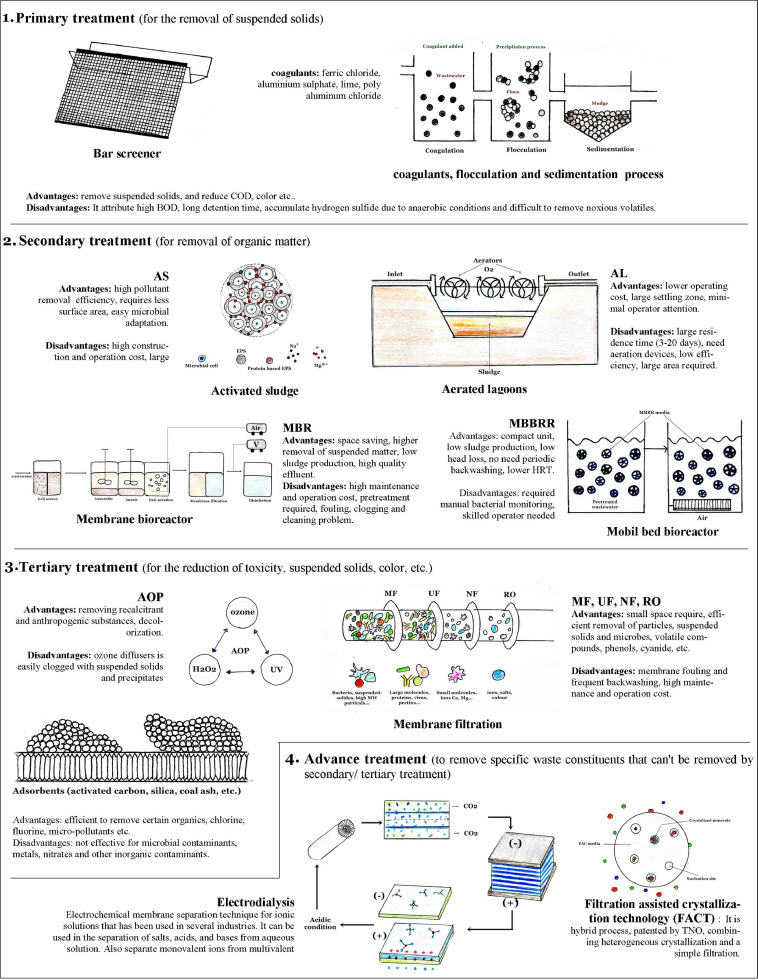
Conventional and advanced physicochemical, biological methods used to treat pulp and paper mill wastewater.

Different methods such as physical, chemical, and biological treatments (AzadiAghdam et al., 2016) have been used to treat industrial wastewater, and each method has its advantage and drawbacks (Fig. 2). The presence of wood components such as cellulose, hemicellulose, and lignin in wastewater is easily degraded via natural processes but due to the incorporation of xenobiotic compounds, it will be hard to biodegrade. The biodegradability index of industrial wastewater is less than 0.4, which means this wastewater is challenging to treat by the biochemical process (Kalyani et al., 2009). Earlier several techniques have been applied to treat pulp and paper industries effluent. Removal of chlorinated organics and chromophoric compounds was attempted by fly ash adsorbing medium (Sell et al., 1994), total organic carbon (TOC), adsorbable organic halides (AOX) and chemical oxygen demand (COD) reduction using photocatalytic treatment (Torrades et al., 2001; Moiseev et al., 2004), 99% AOX reduction was achieved by ultrafiltration treatment (Yao et al., 1994). The membrane-based reverse osmosis and nanofiltration are efficiently used to remove AOX, salt and color (Savant et al., 2006). AOX, salt and color etc., is also reduced by the ozonation treatment followed by biodegradation in a biofilm reactor (Mobius and Helble, 2004). Ganjidoust et al. (1997) used chitosan as a coagulant, which reduces 90% and 70% of color and TOC, respectively.

Among the above-mentioned physicochemical methods, the coagulation/flocculation technique has been the most commonly used in pulp and paper industries to separate suspended and dissolved solids from the wastewater (Toczyłowska-Mamińska, 2017). However, these physical, chemical and electrochemical wastewater treating processes are extravagant when applied alone at large-scale operations. In the coagulation technique, a large amount of toxic sludge is produced, which creates disposal problems. Whereas, added metal (e.g., aluminum) in treated water, resulting in human health implications (Toczyłowska-Mamińska, 2017). Moreover, an extreme pH range is used for optimum treatment, and therefore it's necessary to be neutralize to neutral pH before reuse and recycle (Hubbe et al., 2016; Bajpai, 2018). Oxidation via ozone and hydrogen peroxide is expensive, and oxidation using chlorine species generates secondary pollutants. These physicochemical processes are also responsible for emitting greenhouse gas. Membrane-based technology also faces the flux decline process due to membrane fouling (Lin et al., 2012). Therefore it is necessary to develop economical and eco-friendly methods for the removal of hazardous compounds.

### Biological process

Microbes have the unique capability to convert waste effluents into energy and raw materials for their growth (Ghosh and Thakur, 2017). Moreover, biological processes have cost-effective and eco-friendly compared to the physicochemical process. Commonly, the biological processess were applied after the preliminary clarification treatment. Biological treatment includes the application of bacteria, fungi, and their enzymes as single or in consortium with the various conventional (aerobic, anaerobic, and combination treatments) processes for the removal of organic (lignin-rich) pollutants (Pokhrel and Viraraghavan, 2004; Bajpai, 2018; Chaudhry and Paliwal, 2018). Activated sludge and anaerobic treatment have been widely used in a majority of pulp and paper mills around the world. However, this both conventional biological treatment required large space, high operational cost, generate high amount of sludge etc., which makes overall treatment costly. Therefore, recently emerged MBR and MBBR techniques for wastewater treatment gives first choice over the conventional biological treatments (Iorhemen et al., 2016). This MBR and MBBR techniques required small space, less sludge production, and also provide water reuse option. The various microbial species were reported for pulp and paper mill wastewater treatment are summarized in Table 2. These biological methods are helping to reduce pollution in an eco-friendly way. Pulp and paper wastewater contains the higher molecular weight of cyclic groups of lignin residue, cellulose, and other organic compounds, making it difficult to degrade by microbial degradation (AzadiAghdam et al., 2016). However, microbes have developed a unique strategy to defeat this restriction for complex lignin resides. The use of consortium is a worth-explore method to reduce the organic compounds load in the effluent.

**Table 2:** Microbial species involved in the treatment of pulp and paper mill wastewater.

Microbial species	Pollution parameters	Refs.
Bacteria		
*Aeromonasformicans*	lignin (78%), COD (71%), color (86%)	Gupta et al. (2001)
*Pseudomonas fluorescens*	lignin (45%), COD (79%), phenol (66%), color (75%)	Chauhan and Thakur (2002)
*Paenibacillus* sp., *Aneurinibacillusaneurinilyticus*, and *Bacillus* sp.	lignin (28–53%), COD (52–78%), BOD (65–82%), total phenol (64–77%), color (39–61%)	Raj et al. (2007)
*Serratiamarcescens, Citrobacter* sp., and *Klebsiella* *Pneumonia*	COD (83%), BOD (74%), color (85%)	Chandra et al. (2011)
*Brevibacillusparabrevis*MTCC 12,105	lignin (42.6%), COD (60.3%), color (51.6%)	Hooda et al. (2018)
*Planococcus* sp. TRC1	lignin (74%), COD (85%), phenol (81%), color (96%)	Majumdar et al. (2019)
Fungi		
*Meruliusaureus*and *Fusariumsambucinum*	lignin (79%), COD (89.4%), color (78.6%)	Malaviya and Rathore (2007)
*Cryptococcus* sp.	lignin (24%), color (27%)	Singhal and Thakur (2009)
*Phanerochaetechrysosporium*	lignin (79%), COD (89.4%), color (78.6%)	Saritha et al. (2010)
*Trametespubescens*	2-Chlorophenol (73.39%),2,4-Dichlorophenol (69.59),2,4,6,-Trichlorophenol (38.17), Pentachlorophenol (58.57)	Gonzalez et al. (2010)
*Phanerochaetechrysosporium*	lignin (71%), COD (56%), color (86%)	Chopra and Singh (2012)
*Trametesversicolor*	COD (82%), pentachlorophenol (98%), 2,4,5-trichlorophenol (92%), 3,4-dichlorophenol (90%), 4-chlorophenols (99%), color (80%)	Pedroza-Rodríguez and Rodríguez- Vázquez (2013)
*Bierkanderaadusta*and *Phenarochetecrysosporium*	lignin (74–97%) TOC (35%)	Costa et al. (2017)
*Pleurotusostreatus*	lignin (37.7–46.5%)	Li et al. (2019)
*Pleurotusostreatus*EB016	COD (99.2%), phenol (92.2%)	Heinz et al. (2019)
Algae		
*Chlorella, Chlorococcum, Chlamydomonas, Pandorina, eudorina, Nitzschai, Cyclotella, Microcyctis*, and *Anabaena*	COD (85%), color (75%), AOX (93%)	Tarlan et al. (2002b)
*Scenedesmus* sp.	COD (75%), BOD (82%)	Usha et al. (2016)

#### Aerobic process

Many anaerobic processes are being used to treat large scale pulp and paper industrial wastewater. Extensive lab and large commercial scale research are available for aerobic processes in wastewater treatment. The activated sludge process can reduce the amount of BOD, COD, total suspended solids (TSS), total organic carbon (TOC), AOX, and chlorinated compounds from the pulp and paper wastewater (Ashrafi et al., 2015). Ghoreishi and Haghighi (2007) used Activated sludge (AS) system and determined the capacity to treat pulp and paper wastewater. Although this study showed the AS system's capability to remove 92% COD, 99% BOD, and 97% TSS from the wastewater. Leiviskä et al. (2008) treated wastewater from a pulp and paper mill using AS process and successfully removed 60–70% COD, 95% BOD, and 60% TOC. Similarly, Bengtsson et al. (2008) used the AS process in a batch system and removed 74–95% COD from the pulp and paper mill. Abedinzadeh et al. (2018) carried out AS process using a sequence batch reactor (SBR) in combination with advanced oxidation processes (AOPs) at a bench scale and successfully remove 74.8% COD and 58.3% color. Furthermore, they also enhanced COD and color removal efficiency by Fenton oxidation as post-treatment. Bryant (2010) reported aerated stabilization basins (ASB's) efficiency with nitrogen supplement conditions to remove 67% COD and 90% BOD from the pulp and paper wastewater. Other investigations (Dykstra et al., 2015; Lewis et al., 2018) treated various pulp and paper wastewater using the ASB's process and could remove 84–88% COD, 90–94% BOD, 82–94% phytosterol and other AOX and chlorinated compounds. The aerobic process required a higher amount of oxygen to promote biological oxidation. All these aerobic treatments have a significant disadvantage because of the constant requirement of high oxygen and energy supply (Toczyłowska-Mamińska, 2017).

#### Anaerobic process

In anaerobic processes, up-flow anaerobic sludge blanket reactor (UASBR) and fluidized-bed reactor (FBR) have been widely used and established methods to treat pulp and paper wastewaters (Ashrafi et al., 2015). The anaerobic process has greater COD removal capacity in a small process area. Chinnaraj and Venkoba Rao (2006) utilized the UASB reactor and effectively removed COD (80–93%) from the agro-based pulp and paper industry's wastewater. This UASB reactor technique has an advantage over the anaerobic lagoon system because a UASB reactor has a methane recovery system, and this methane is used as biogas or fuel. The UASB reactors used by Buzzini and Pires (2007) for the treatment of bleached and unbleached kraft industry wastewaters. These studies showed 78–82% COD and 71–99% chlorinated organics compounds removal from wastewater. According to Ortega-Clemente and Poggi-Varaldo (2007), adding ligninolytic fungi in an FBR to treat pulping wastewater improves the COD and color removal efficiencies. Deshmukh et al. (2009) treated bleaching wastewater using an up-flow anaerobic filter (UAF), and effectivety removes COD (50%), BOD (70%), and AOX (50%). Detailed studies revealed that the UASB reactor had lower energy requirements, the fixed-film reactor had lower capital cost, and FBR has higher contaminant removal efficiency in treating pulp and paper wastewater (Rajeshwari et al., 2000).

#### Role of bacteria

In pulp and paper effluent treatment, bacteria play an essential role due to their vast environmental adaptability. Bacterial genus such as *Bacillus, Pseudomonas, Micrococcus, Methylobacterium, Ancylobacter, Paenibacillus*, etc., was reported decolorizing, lignin removing, and organochlorine degradation ability from the pulp and paper industries wastewater (Chaudhry and Paliwal, 2018). Bacterial strain *B. megaterium, P. aeruginosa, Paenibacillus* sp., *Aneurinibacillus aneurinilyticus, Pseudochrobactrum glaciale, Providencia rettgeri, Pantoea* sp., were reported for their decolourizing ability from the bleach kraft effluent (Raj et al., 2007; Tiku et al., 2010; Chandra and Singh, 2012). Apart from the decolourizing ability, bacteria have a piece of unique enzymatic machinery that offers lignin's depolymerization. The bacterial species *B. subtilis, Micrococcus luteus, Cupriavidus basilensis,* etc., were reported to degrade lignin from the pulp and paper wastewater (Tyagi et al., 2014). *B. aryabhattai* reduced 67% and 54% color and lignin respectively from the pulp and paper mill effluent (Zainith et al., 2019). Degradation of organochlorine from the bleached kraft pulp and paper industries wastewater by strains of *Pseudomonas, Ancylobacter*, and *Methylobacterium* was reported by Keharia and Madamwar (2003), and they observed that *Ancylobacter* has the potential to reduce the AOX from softwood effluents. The lignin degradation by bacteria is limited because lignin-degrading enzymes such as laccases, xylanases, manganese-dependent peroxidase, glutathione S-transferases, ring cleaving dioxygenases, monooxygenases, and phenol oxidases were produced in a lesser quantity (Paliwal et al., 2012). Most bacteria can degrade the low-molecular-weight lignin components, which produce after the fungal attack on lignin. Moreover, the single bacterial strain has a lack all the lignin-degrading enzymes. Therefore, applying more than two bacterial strains or synergy work with fungi is necessary, which helps complete lignin removal from the pulp and paper industries wastewater.

#### Role of fungi

Fungi are the potent biological agents to depolymerize lignin molecules non-specifically. Fungi have a unique extracellular enzyme system, including laccase, lignin peroxidase, manganese peroxidase, which is responsible for decolorization, and lignin depolymerization. Fungi also have a high survival rate in the high effluent load (Kamali and Khodaparast, 2015). Fungal strains have also shown superior resistance against inhibitory compounds than do bacterial species. The cell walls of fungi are made by an extra-polysaccharide matrix, which helps them from inhibitory compounds through adsorption. Additionally, fungi contain more genes for tolerating inhibitory compounds than bacteria, which might help to adapt to the hazardous environment (Gupta and Gupta, 2019). All these extraordinary features make fungi a potential candidate for the biodegradation of pulp and paper effluent. The previous study showed that certain fungi could degrade the complex organochlorine compounds and absorb heavy metals from aqueous solution (Bajpai, 2018). Several fungal species such as *Aspergillus niger*, Trametes versicolor, *Coriolus versicolor*, Phanerochaete chrysosporium, *Ganoderma lucidum, Irpex lacteus, Fomes lividus, Lentinus edodes, Schizophyllum commune, Tinctoria borbonica, Trichoderma* sp., *Datronia* sp., *Thelephora* sp., *Pleurotus* sp., and *Ceriporiopsis* sp. have been identified as significant lignin degrader (Singh, 2018). Among the other fungi, white-rot fungi, namely T. versicolor and P. chrysosporium are well-characterized for their degrading and decolorizing nature. These white-rot fungi have been evaluated on a pilot scale using various strategies like trickling filters, airlift reactors, and fluidized bed reactors (Bajpai, 2018). Detail study on *P. chrysosporium* showed that mycelia have color removal and organochlorine degrading efficiency (Pointing, 2001). *Datronia* sp. KAPI0039 (54.9%) and *Trichaptum* sp. KAPI0025 (54.4%) were reported to decolourize the pulp and paper mill wastewater (Apiwattanapiwat et al., 2006). *Pleurotus sajorcaju, Rhizopus oryzae, Perenniporia tephropora* KU-Alk4, and *Phlebia brevispora* BAFC 633 reported for biodegradation of organic compounds of bleached pulp wastewater (Fonseca et al., 2015; Singh and Arya, 2019).

#### Role of algae

Remediation with algae is another approach to treat pulp and paper industrial polluted wastewater. The algae can transform/decolourize the chromophoric lignin molecules into a colorless form via its metabolic activity (Chandra and Singh, 2012). Several algal species have been reported for their ability to degrade/remove kraft lignin and heavy metals from the wastewater of paper industries (Table 2). The algal species Planktochlorella nurekis and Chlamydomonas reinhardtii were reported to effectively remove nitrate, phosphate, COD, and many metals from the pulp and paper wastewater (Sasi et al., 2020). It is found that mixed algal strains have better degradation than a shorter time to a single algal species, which takes more time. Tarlan et al. (2002a) apply a hybrid algal approach using Chlorella, Chlorococcum, Chlamydomonas, Pandorina, and eudorina, resultant 75% COD, 84% color, and 80% AOX was removed. Usha et al. (2016) carried out wastewater treatment by employing the mixed culture of Scenedesmus sp. They found maximum removal of BOD and COD in a lab-scale study.

#### Role of enzymes

Microbial enzymes include cellulases, xylanases, laccases, peroxidases, catalases, amylases, proteases, lipases, etc., which play an essential role in managing the organic waste of pulp and paper industries. The rate of waste bioremediation depends on the microbial species and environmental conditions. The white-rot fungi produce one or more types of ligninolytic enzymes reported to degrade lignin and various kinds of xenobiotic compounds (Deshmukh et al., 2016). The ligninolytic enzymes produced by white-rot fungi are characterized into two groups: Lignin Peroxidases (LiP) and Manganese Peroxidases (MnP) (He et al., 2015). Laccase and class II peroxidases from white-rot fungi are well established to degrade persistent organic pollutants (Ikehata, 2015). Laccase oxidizes most phenolic and non-phenolic compounds and *T. versicolor* reported more than 20 times higher laccase activity than other microbes (Margotet al., 2013). Enzymatic treatment is entirely or partially removed the pollutant compounds such as pentachlorophenol (PCP), 4,5-dichloroguaicol, 4,5,6-trichloroguaicol, tetrachloroguaiacol, pentachlorophenol, and 2,4,6-trichlorophenol from the pulp and paper industry wastewater (Shankar et al., 2020). According to Hossain and Ismail (2015) study, laccase has the potential to decolourize the black liquor and also reduce reported BOD and COD of the pulp and paper wastewater. However, the study showed that the waste degradation efficiency is improving by immobilizing the microbial enzymes.

## Membrane bioreactor technology

The membrane bioreactor (MBR) technologies are commonly used for the biodegradation and physical separation of waste compounds. This MBR technology involves the fusion of the biological reactor, which is coupled with membrane units. In this technology, membranes are used to critical solid-liquid separation. Researchers have already reported this MBR technology for treating various kinds of wastewater, including municipal, high strength wastewater, pharmaceutical, tannery, food industry, dye industry, etc. (Izadi et al., 2018). Beside this, MBR technology was also recognized for the production of clarified and high-quality treated effluent. MBR technology comparatively more advantages over the activated sludge method due to the less sludge production, higher separation efficiency, and retaining low molecular weight organic micropollutants (Neoh et al., 2016). Further, MBR also offers wastewater reuse in various industrial and agricultural sectors (Krzeminski et al., 2017; Patel and Patel, 2020). Beside the advantage, membrane fouling is a significant drawback in MBR technology. Due to the pore-clogging, a foulant layer is formulated, reflecting the negative impact on MBR performance. Low filterability/high capillary suction time is also a major concern to limit MBR use in wastewater treatment (Scholes et al., 2019). There is an insufficient source of published literature regarding long-term operational concerns in full-scale industrial MBR. Therefore, environmental researchers are currently focusing on improving the MBR technology by coupling them with advanced oxidation processes, reverse and forward osmosis, granulation technology, membrane distillation bioreactor (MDBR), and hybrid moving bed biofilm reactor-membrane bioreactor (Hybrid MBBR-MBR). Studies have shown that this integrated technique overcomes the membrane fouling problem by regular back-washing membrane pores and enhances the overall stability of treatment. Qu et al. (2012) achieve an 88.6 ± 1.9 to 92.3 ± 0.7% COD reduction by completely decolorizing the effluent by combining thermophilic submerged aerobic membrane bioreactor and electrochemical oxidation technology. Merayo et al. (2013) reported 90% COD removal of pulp mill effluent by MBR integrated with advanced oxidation processes (AOPs) followed by ozonation. The sequence batch reactor incorporated with the bacterial consortium (*Klebsiella* sp., *Alcaligens* sp. and *Cronobacter* sp.) reduce the 72.3% COD, 91.1% BOD, 55% color, 45.4% AOX, 22% TDS, and 86.7% TSS within 14 h from the wastewater of paper mill (Kumar et al., 2014).

Giacobbo et al. (2015) designated the integrated MBR-photoelectrooxidation (MBR–PEO) method for tannery wastewater treatment, and they achieved a 97% COD and 87.8% BOD reduction with MBR–PEO reactor. The integration of MBR and electrocoagulation is highly efficient in removing more than 90% metals, i.e. Cu, Cr and Zn (Vijayakumar and Balasubramanian, 2015). The MBR integrating with AOPs and electrocoagulation techniques helped overcome membrane fouling, removal of recalcitrant and coloured compounds, and metals. In biofilm membrane bioreactor (BF-MBR), addition carriers are incorporated inside the MBR, which is responsible for decreasing the concentration of suspended solids without limiting the process's efficiency and leads to mitigation of membrane fouling (Neoh et al., 2016). Gao et al. (2016) reported 83% of COD removal from the pulping wastewater by a submerged anaerobic membrane bioreactor (SAnMBR). Izadi et al. (2019) used a fixed-bed membrane bioreactor (FBMBR) with a hydraulic retention time (HRT), which reduce COD (92–99%), ammonium (59–97%), nitrite (78–97%), nitrate (59–98%) and total nitrogen (62–92%). Similarly, a hybrid airlift membrane bioreactor (HAMBR) was developed by Izadi et al. (2020), was effectively reduce COD (88–99%), ammonium (54–83%), nitrite (70–90%), nitrate (65–95%) and total nitrogen (61–90%). Recently documented submerged polyvinylidene fluoride MBR by Poojamnong et al. (2020) having 73% COD, and 79% color removal efficiency from the pulp and paper industry wastewater.

## Moving-bed biofilm reactor (MBBR) technology

The biofilm-based MBBR technology was invented in the late 1980s to treat wastewater (Ødegaard, 2006). Different biofilm systems were already used in trickling filters, granular media biofilters, rotating biological contractors, etc., for wastewater treatment, but these methods have several disadvantages. Therefore, the MBBR process emerged and was established as a simple, compact, and flexible wastewater treatment. More than 700 MBBR based wastewater treatment systems are operated/installed in over 50 countries (Ødegaard, 2006). The MBBR process has shown great potential to reduce suspended solids with the production of high-quality reusable water.

The MBBR system comprises an aeration tank with special design carriers that are made of plastic (Fig. 2). The microbial decomposers adhere to carriers and are responsible for the formation of biofilm (De Oliveira et al., 2014). These carriers increase surface area for microbial growth and also improve cell retention time. The higher concentration of solids adhered with carriers that make fast decomposition of organic matter. The significant advantage of these processes including the separation of surplus biomass without the sludge recirculation process. The MBBR process has high treatment efficiency, low operational, capital, maintenance, and replacement cost (Barwal and Chaudhary, 2014). In comparision to MBR technology, there is no membrane surface fouling and membrane channel clogging problems in MBBR technology. Whereas, the major cons associated with this technology is to manual monitoring i.e., periodically sample collection and analyses for the presence of microbes on the carriers. Different types of insects like sewage flies, mosquitoes and red worms are also attracted toward the biofilm.

The MBBR system has been successfully used to treat municipal and industrial wastewater, including pulp and paper industries, pharmaceutical, dairy, refinery and slaughterhouse (Barwal and Chaudhary, 2014). Both pilot-scale and full-scale MBBR plants have been documented to treat releasing industrial wastewater (Ødegaard, 2006). Papermill of Klabin and Suzano (Brazil), Stora Papyrus Grycksbo AB, Stora Cell Industri AB, StoraForsBillerud AB and Norske Sande Skog (Sweden) are documented for treating the wastewater via the MBBR process (Rusten et al., 1994). Broch-Due et al. (1994) carried out a pilot-scale study using the MBBR system, removing 98% toxicity and 70% COD at organic load 25 kg COD m^−3^ day^−1^ from the paper mill wastewater. Wastewater of integrated newsprint mill was treated by pilot-scale MBBR system, resultant 65–75% COD and 85–95% BOD removal at 4–5 h HRT (Broch-Due et al., 1997). Embley (2001) successfully treated kraft pulp wastewater using the MBBR system, removing 63% BOD_5_. Jahren et al. (2002) reported 60–65% soluble chemical oxygen demand (SCOD) removal at 2.5–3.5 kg SCOD m^−3^ d^−1^ organic loading rates of the thermo-mechanical pulping white-water using lab-scale MBBR system. Das and Naga (2011) treated combine effluents of the pulp mill, powerhouse, chemical recovery plant and domestic via a full-scale MBBR system and removed 50% SCOD, 21.53% COD and 33.5% BOD. The modified MBBR process has more advantages over the traditional process. The paper mill SuzanoPapele Cellulose (Mucuri/Brazil) carried out the treatment in aerated lagoon followed by three MBBR in series with HRT system. These combined processes help to enhance the wastewater treatment process in a shorter time (Oliveira et al., 2014). Leyva-Diaz et al. (2013) reported 90–91% COD removal from the wastewater treatment plant using the MBBR-MBR system. A novel anaerobic MBBR-MFC was recently designed for simultaneous bioelectricity generation and paper mill wastewater treatment (Chen et al., 2020). These novel approaches showed superior bioelectricity performance (power density: 94.5 mW/m^2^; internal resistance 35.7 Ω) with 65.6% COD removal efficiencies.

## Recycle and reuse biologically treated wastewater

Due to the high water demands in the pulp and paper industries, it is necessary to recycle and reuse generated wastewater. The resue of untreated water can increase the concentration of organic and inorganic matter, which affect the paper quality and also increase corrosion and odours (Thompson et al., 2001). The coupling of membrane filtration with existing wastewater treatment plant can help to enhance the efficiency of overall treatment and also offer the reuse of wastewater. Chen and Horan (1998) treated wastewater of activated sludge treatment plants with chemical coagulation to produce high-grade recycled water suitable for reuse in fiber plants. The microbial treatment or combine physicochemical process can effectively reduce the high concentration of toxic pollutants and improves wastewater quality. The microfiltration followed by the reverse osmosis filtration step enhances the recovery and reuse of more than 80% of the original wastewater (Pizzichini et al., 2005).

Similarly, Sahinkaya et al. (2008) treat and reuse denim textile wastewater by coupling the activated sludge treatment with nanofiltration, which has 91 ± 2% COD and 75 ± 10% color removal efficiency. The lab-scale study carried out by Mänttäri et al. (2008) suggested that the use of nanofiltration after microbial treatment reduce the contaminants (turbidity, color, COD, sulfate, chloride, and bicarbonate) from wastewater and open the door for reuse. The integrated thermophilic submerged aerobic membrane bioreactor (TSAMBR), followed by the electrochemical oxidation process, produces high-quality water that is further reused in various stages (Qu et al., 2012). With this strategy, complete decolourization was achieved, and 96.2 ± 1.2 to 98.2 ± 0.3% COD was removed.

## Emerging technologies

There is rapid improvement in the new technology/process development due to the necessity of treating pulp and paper industry wastewater. Combining the micro-physicochemical process, modification, or hybrid of the existing process will reduce the contaminants and improve treated water quality for reuse. Advance technology (Fig. 2), such as biosorption, photoelectrolysis, advanced oxidation (Photo-Fenton oxidation), Filtration Assisted Crystallization Technology (FACT) etc., are in the initial development stages (Crini and Lichtfouse, 2019).

## Conclusions

Based on the pulping process, pulp and paper industry wastewater contains various complex organic and inorganic compounds that are generally considered toxic for all living biota. Physicochemical treatment is usually applied to treat pulp and paper wastewater, but it's economically unsuitable, whereas biological process takes longer time, and alone this process straggles to treat wastewater. Hybrid MBR technology has emerged with solid potential to treat wastewater. These hybrid MBR and filtration technology provide the option of recycling and reuse of pulp and paper wastewater. Similarly, MBBR-MFCs are another attractive technology that can simultaneously treat wastewater and convert chemical energy to electricity in one step. Reducing freshwater consumption is also necessary, and it is achieved by blending treated wastewater with fresh water. Reuse of wastewater overcame the large consumption of fresh water and an essential economic and ecological point of view.

## CRediT authorship contribution statement

**Kartik Patel:** Conceptualization, Writing – original draft. **Niky Patel:** Project administration. **Nilam Vaghamshi:** Data curation. **Kamlesh Shah:** Data curation. **Srinivas Murthy Dugdirala:** Data curation. **Pravin Dudhagara:** Methodology.

## Declaration of Competing Interest

There are no conflicts of interest among the authors.

## References

[bib0001] Abedinzadeh N., Shariat M., Monavari S.M., Pendashteh A. (2018). Evaluation of color and COD removal by Fenton from biologically (SBR) pre-treated pulp and paper wastewater. Process. Saf. Environ. Prot..

[bib0003] Apiwatanapiwat W., Siriacha P., Vaithanomsat P. (2006). Screening of fungi for decolorization of wastewater from pulp and paper industry. Agric. Nat. Resour..

[bib0004] Ashrafi O., Yerushalmi L., Haghighat F. (2015). Wastewater treatment in the pulp-and-paper industry: a review of treatment processes and the associated greenhouse gas emission. J. Environ. Manag..

[bib0005] AzadiAghdam M., Kariminia H.R., Safari S. (2016). Removal of lignin, COD, and color from pulp and paper wastewater using electrocoagulation. Desalin.Water Treat..

[bib0006] Bajpai P. (2018). Biotechnology for Pulp and Paper Processing.

[bib0007] Barwal A., Chaudhary R. (2014). To study the performance of biocarriers in moving bed biofilm reactor (MBBR) technology and kinetics of biofilm for retrofitting the existing aerobic treatment systems: a review. Rev. Environ. Sci. Biotechnol..

[bib0008] Bengtsson S., Werker A., Christensson M., Welander T. (2008). Production of polyhydroxyalkanoates by activated sludge treating a paper mill wastewater. Bioresour. Technol..

[bib0009] Broch-Due A., Andersen R., Kristoffersen O. (1994). Pilot plant experience with an aerobic moving bed biofilm reactor for treatment of NSSC wastewater. Water Sci. Technol..

[bib0010] Broch-Due A., Andersen R., Opheim B. (1997). Treatment of integrated newsprint mill wastewater in moving bed biofilm reactors. Water Sci. Technol..

[bib0011] Bryant C.W. (2010). Updating a model of pulp and paper wastewater treatment in a partial-mix aerated stabilization basin system. Water Sci. Technol..

[bib0012] Buzzini A.P., Pires E.C. (2007). Evaluation of aupflow anaerobic sludge blanket reactor with partial recirculation of effluent used to treat wastewaters from pulp and paper plants. Bioresour. Technol..

[bib0013] Cabrera M.N. (2017). Pulp mill wastewater: characteristics and treatment. Biol. Wastewater Treat. Resour. Recovery.

[bib0014] Chandra R., Singh R. (2012). Decolourisation and detoxification of rayon grade pulp paper mill effluent by mixed bacterial culture isolated from pulp paper mill effluent polluted site. Biochem. Eng. J..

[bib0015] Chandra R., Abhishek A., Sankhwar M. (2011). Bacterial decolorization and detoxification of black liquor from rayon grade pulp manufacturing paper industry and detection of their metabolic products. Bioresour. Technol..

[bib0016] Chaudhry S., Paliwal R. (2018).

[bib0017] Chauhan N., Thakur I.S. (2002). Treatment of pulp and paper mill effluent by pseudomonas fluorescens in fixed film bioreactor. Pollut. Res..

[bib0018] Chen F., Zeng S., Luo Z., Ma J., Zhu Q., Zhang S. (2020). A novel MBBR–MFC integrated system for high-strength pulp/paper wastewater treatment and bioelectricity generation. Sep. Sci. Technol..

[bib0019] Chen W., Horan N.J. (1998). The treatment of a high strength pulp and paper mill effluent for wastewater reuse: III) tertiary treatment options for pulp and paper mill wastewater to achieve effluent recycle. Environ. Technol..

[bib0020] Chinnaraj S., Rao G.V. (2006). Implementation of an UASB anaerobic digester at bagasse-based pulp and paper industry. Biomass Bioenergy.

[bib0021] Chopra A.K., Singh P.P. (2012). Removal of color, COD and lignin from pulp and paper mill effluent by phanerochaetechrysosporium and aspergillusfumigatus. J. Chem. Pharm. Res..

[bib0022] Costa S., Dedola D.G., Pellizzari S., Blo R., Rugiero I., Pedrini P., Tamburini E. (2017). Lignin biodegradation in pulp-and-paper mill wastewater by selected white rot fungi. Water.

[bib0023] Crini G., Lichtfouse E. (2019). Advantages and disadvantages of techniques used for wastewater treatment. Environ. Chem. Lett..

[bib0024] Dahlman O.B., Reimann A.K., Stromberg L.M., Morck R.E. (1995). High-molecular-weight effluent materials from modern ECF and TCF bleaching. TAPPI J. (USA).

[bib0025] Das, A., Naga, R.N., 2011. Activated sludge process with MBBR technology at ETP. I.P.P.T.A.J, 23, 135–137. 10.1007/s10311-018-0785-9.

[bib0026] De Oliveira D.V.M., Rabelo M.D., Nariyoshi Y.N. (2014). Evaluation of a MBBR (moving bed biofilm reactor) pilot plant for treatment of pulp and paper mill wastewater. Int. J. Environ. Monit. Anal..

[bib0027] Deshmukh R., Khardenavis A.A., Purohit H.J. (2016). Diverse metabolic capacities of fungi for bioremediation. Indian J. Microbiol..

[bib0028] Dykstra C.M., Giles H.D., Banerjee S., Pavlostathis S.G (2015). Fate and biotransformation of phytosterols during treatment of pulp and paper wastewater in a simulated aerated stabilization basin. Water Res..

[bib0029] Embley D. (2001). Proceedings of the TAPPI International Environmental, Health and Safety Conference and Exhibit.

[bib0030] Eskelinen K., Särkkä H., Kurniawan T.A., Sillanpää M.E. (2010). Removal of recalcitrant contaminants from bleaching effluents in pulp and paper mills using ultrasonic irradiation and Fenton-like oxidation, electrochemical treatment, and/or chemical precipitation: a comparative study. Desalination.

[bib0031] Fonseca M.I., Farina J.I., Sadanoski M.A., D'Errico R., Villalba L.L., Zapata P.D. (2015). Decolorisation of Kraft liquor effluents and biochemical characterization of laccases from Phlebiabrevispora BAFC 633. Int. Biodeterior. Biodegrad..

[bib0032] Ganjidoust H., Tatsumi K., Yamagishi T., Gholian R.N. (1997). Effect of synthetic and natural coagulant on lignin removal from pulp and paper wastewater. Water Sci. Technol..

[bib0033] Gao W.J., Han M.N., Xu C.C., Liao B.Q., Hong Y., Cumin J., Dagnew M. (2016). Performance of submerged anaerobic membrane bioreactor for thermomechanical pulping wastewater treatment. J. Water Process. Eng..

[bib0034] Garg S.K., Tripathi M., Kumar S., Singh S.K., Singh S.K. (2012). Microbial dechlorination of chloroorganics and simultaneous decolorization of pulp–paper mill effluent by Pseudomonas putida MTCC 10510 augmentation. Environ. Monit. Assess..

[bib0035] Ghoreishi S.M., Haghighi M.R. (2007). Chromophores removal in pulp and paper mill effluent via hydrogenation-biological batch reactors. Chem. Eng. J..

[bib0036] Ghosh P., Thakur I.S. (2017). Developments in Fungal Biology and Applied Mycology.

[bib0037] Giacobbo A., Feron G.L., Rodrigues M.A.S., Ferreira J.Z., Meneguzzi A., Bernardes A.M. (2015). Integration of membrane bioreactor and advanced oxidation processes for water recovery in leather industry. Desalin.Water Treat..

[bib0039] Gupta A., Gupta R. (2019). Advances in Biological Treatment of Industrial Waste Water and their Recycling for a Sustainable Future.

[bib0040] Gupta V.K., Minocha A.K., Jain N. (2001). Batch and continuous studies on treatment of pulp mill wastewater by aeromonasformicans. J. Chem. Technol. Biotechnol..

[bib0041] Haq I., Raj A. (2020). Bioremediation of Industrial Waste For Environmental Safety.

[bib0042] He L., Huang H., Zhang Z., Lei Z. (2015). A review of hydrothermal pretreatment of lignocellulosic biomass for enhanced biogas production. Curr. Org. Chem..

[bib0043] Heinz O.L., Cunha M.A., Amorim J.S., Barbosa-Dekker A.M., Dekker R.F., Barreto-Rodrigues M. (2019). Combined fungal and photo-oxidative Fenton processes for the treatment of wood-laminate industrial waste effluent. J. Hazard. Mater..

[bib0044] Hooda R., Bhardwaj N.K., Singh P. (2018). Brevibacillus parabrevis MTCC 12105: a potential bacterium for pulp and paper effluent degradation. World J. Microbiol. Biotechnol..

[bib0045] Hossain K., Ismail N. (2015). Bioremediation and detoxification of pulp and paper mill effluent: a review. Res. J. Environ. Toxicol.

[bib0046] Hubbe M.A., Metts J.R., Hermosilla D., Blanco M.A., Yerushalmi L., Haghighat F.L., Haghighat F., Lindholm-Lehto P., Khodaparast Z., Kamali M., Elliott A. (2016). Wastewater treatment and reclamation: a review of pulp and paper industry practices and opportunities. BioResources.

[bib0047] Ikehata, K.,2015.Use of fungal laccases and peroxidases for enzymatic treatment of wastewater containing synthetic dyes. In Green Chemistry for Dyes Removal from Wastewater. 10.1002/9781118721001.ch6.

[bib0048] Iorhemen O.T., Hamza R.A., Tay J.H. (2016). Membrane bioreactor (MBR) technology for wastewater treatment and reclamation: membrane fouling. Membranes.

[bib0049] Izadi A., Hosseini M., Darzi G.N., Bidhendi G.N., Shariati F.P. (2019). Performance of an integrated fixed bed membrane bioreactor (FBMBR) applied to pollutant removal from paper-recycling wastewater. Water Resour. Ind..

[bib0050] Izadi A., Hosseini M., NajafpourDarzi G., NabiBidhendi G., PajoumShariati F., Mosaddeghi M.R. (2018). Perspectives on membrane bioreactor potential for treatment of pulp and paper industry wastewater: a critical review. J. Appl. Biotechnol. Rep..

[bib0051] Izadi A., Hosseini M., PajoumShariati F., NajafpourDarzi G., NabiBidhendi G. (2020). Treatment of real paper-recycling wastewater in a novel hybrid airlift membrane bioreactor (HAMBR) for simultaneous removal of organic matter and nutrients. Iran. J. Chem. Chem. Eng..

[bib0052] Jahren S.J., Rintala J.A., Ødegaard H. (2002). Aerobic moving bed biofilm reactor treating thermomechanical pulping white-water under thermophilic conditions. Water Res..

[bib0053] Kalyani K.P., Balasubramanian N., Srinivasakannan C. (2009). Decolorisation and COD reduction of paper industrial effluent using electrocoagulation. Chem. Eng. J..

[bib0054] Kamali M., Khodaparast Z. (2015). Review on recent developments on pulp and paper mill wastewater treatment. Ecotoxicol. Environ. Saf..

[bib0055] Keharia H., Madamwar D. (2003). Bioremediation concepts for treatment of dye containing wastewater: a review. Indian J. Exp. Biol..

[bib0056] Krzeminski P., Leverette L., Malamis S., Katsou E. (2017). Membrane bioreactors–a review on recent developments in energy reduction, fouling control, novel configurations, LCA and market prospects. J. Membr. Sci..

[bib0057] Kumar D., Gaurav V.K., Sharma C. (2018). Ecofriendly remediation of pulp and paper industry wastewater by electrocoagulation and its application in agriculture. Am. J. Plant Sci..

[bib0058] Leiviskä T., Nurmesniemi H., Pöykiö R., Rämö J., Kuokkanen T., Pellinen J. (2008). Effect of biological wastewater treatment on the molecular weight distribution of soluble organic compounds and on the reduction of BOD, COD and P in pulp and paper mill effluent. Water Res..

[bib0059] Lewis R., Cohen J., Awad J., Burger H., Marzouk J., Burch G., Lewis D.M., van Leeuwen J.A. (2018). Study of the impacts of process changes of a pulp and paper mill on aerated stabilization basin (ASB) performance. Chemosphere.

[bib0060] Leyva-Díaz J.C., Calderón K., Rodríguez F.A., González-López J., Hontoria E., Poyatos J.M. (2013). Comparative kinetic study between moving bed biofilm reactor-membrane bioreactor and membrane bioreactor systems and their influence on organic matter and nutrients removal. Biochem. Eng. J..

[bib0061] Li F., Ma F., Zhao H., Zhang S., Wang L., Zhang X., Yu H. (2019). A lytic polysaccharide monooxygenase from a white-rot fungus drives the degradation of lignin by a versatile peroxidase. Appl. Environ. Microbiol..

[bib0062] Lin H., Gao W., Meng F., Liao B.Q., Leung K.T., Zhao L., Chen J., Hong H. (2012). Membrane bioreactors for industrial wastewater treatment: a critical review. Crit. Rev. Environ. Sci. Technol..

[bib0063] Mahesh S., Garg K.K., Srivastava V.C., Mishra I.M., Prasad B., Mall I.D. (2016). Continuous electrocoagulation treatment of pulp and paper mill wastewater: operating cost and sludge study. RSC Adv..

[bib0064] Majumdar S., Priyadarshinee R., Kumar A., Mandal T., Mandal D.D. (2019). Exploring planococcus sp. TRC1, a bacterial isolate, for carotenoid pigment production and detoxification of paper mill effluent in immobilized fluidized bed reactor. J. Clean. Prod..

[bib0065] Malaviya P., Rathore V.S. (2007). Bioremediation of pulp and paper mill effluent by a novel fungal consortium isolated from polluted soil. Environ. Technol..

[bib0066] Mänttäri M., Kuosa M., Kallas J., Nyström M. (2008). Membrane filtration and ozone treatment of biologically treated effluents from the pulp and paper industry. J. Membr. Sci..

[bib0067] Margot J., Bennati-Granier C., Maillard J., Blánquez P., Barry D.A., Holliger C. (2013). Bacterial versus fungal laccase: potential for micropollutant degradation. AMB Express.

[bib0068] Merayo N., Hermosilla D., Blanco L., Cortijo L., Blanco Á. (2013). Assessing the application of advanced oxidation processes, and their combination with biological treatment, to effluents from pulp and paper industry. J. Hazard. Mater..

[bib0069] Mir-Tutusaus J.A., Baccar R., Caminal G., Sarrà M. (2018). Can white-rot fungi be a real wastewater treatment alternative for organic micropollutants removal? A review. Water Res..

[bib0070] Möbius C.H., Helble A. (2004). Combined ozonation and biofilm treatment for reuse of papermill wastewaters. Water Sci. Technol..

[bib0071] Moiseev A., Schroeder H., Kotsaridou-Nagel M., Geissen S.U., Vogelpohl A. (2004). Photocatalytical polishing of paper-mill effluents. Water Sci. Technol..

[bib0072] Negi R., Suthar S. (2018). Degradation of paper mill wastewater sludge and cow dung by brown-rot fungi Oligoporus placenta and earthworm (Eiseniafetida) during vermicomposting. J. Clean. Prod..

[bib0073] Neoh C.H., Noor Z.Z., Mutamim N.S.A., Lim C.K. (2016). Green technology in wastewater treatment technologies: integration of membrane bioreactor with various wastewater treatment systems. Chem. Eng. J..

[bib0074] Ødegaard H. (2006). Innovations in wastewater treatment:–the moving bed biofilm process. Water Sci. Technol..

[bib0075] Ortega-Clemente A., Marín-Mezo G., Ponce-Noyola M.T., Montes-Horcasitas M.C., Caffarel-Méndez S., Barrera-Cortés J., Poggi-Varaldo H.M. (2007). Comparison of two continuous fungal bioreactors for posttreatment of anaerobically pretreated weak black liquor from kraft pulp mills. Biotechnol. Bioeng..

[bib0076] Paliwal R., Rawat A.P., Rawat M., Rai J.P.N. (2012). Bioligninolysis: recent updates for biotechnological solution. Appl. Biochem. Biotechnol..

[bib0077] Patel K., Dudhagara P. (2020). Compatibility testing and enhancing the pulp bleaching process by hydrolases of the newly isolated thermophilicIsoptericolavariabilis strain UD-6. Biocatal. Biotransform...

[bib0078] Patel K., Patel M. (2020). Improving bioremediation process of petroleum wastewater using biosurfactants producing Stenotrophomonas sp. S1VKR-26 and assessment of phytotoxicity. Bioresour. Technol..

[bib0079] Pedroza-Rodríguez A.M., Rodríguez-Vázquez R. (2013). Optimization of C/N ratio and inducers for wastewater paper industry treatment using Trametesversicolor immobilized in bubble column reactor. J. Mycol..

[bib0080] Pizzichini M., Russo C., Di Meo C. (2005). Purification of pulp and paper wastewater, with membrane technology, for water reuse in a closed loop. Desalination.

[bib0081] Pointing S. (2001). Feasibility of bioremediation by white-rot fungi. Appl. Microbiol. Biotechnol..

[bib0082] Pokhrel D., Viraraghavan T. (2004). Treatment of pulp and paper mill wastewater—a review. Sci. Total Environ..

[bib0083] Poojamnong K., Tungsudjawong K., Khongnakorn W., Jutaporn P. (2020). Characterization of reversible and irreversible foulants in membrane bioreactor (MBR) for eucalyptus pulp and paper mill wastewater treatment using fluorescence regional integration. J. Environ. Chem. Eng..

[bib0084] Qu X., Gao W.J., Han M.N., Chen A., Liao B.Q. (2012). Integrated thermophilic submerged aerobic membrane bioreactor and electrochemical oxidation for pulp and paper effluent treatment–towards system closure. Bioresour. Technol..

[bib0085] Raj A., Kumar S., Haq I., Singh S.K. (2014). Bioremediation and toxicity reduction in pulp and paper mill effluent by newly isolated ligninolyticPaenibacillus sp. Ecol. Eng..

[bib0086] Raj A., Reddy M.K., Chandra R. (2007). Decolourisation and treatment of pulp and paper mill effluent by lignin-degrading bacillus sp. J. Chem. Technol. Biotechnol..

[bib0087] Rajeshwari K.V., Balakrishnan M., Kansal A., Lata K., Kishore V.V.N. (2000). State-of-the-art of anaerobic digestion technology for industrial wastewater treatment. Renew. Sustain. Energy Rev..

[bib0088] Rajwar D., Paliwal R., Rai J.P.N. (2017). Biodegradation of pulp and paper mill effluent by co-culturing ascomycetous fungi in repeated batch process. Environ. Monit. Assess..

[bib0089] Rusten B., Mattsson E., Broch-Due A., Westrum T. (1994). Treatment of pulp and paper industry wastewaters in novel moving bed biofilm reactors. Water Sci. Technol..

[bib0090] Sahinkaya E., Uzal N., Yetis U., Dilek F.B. (2008). Biological treatment and nanofiltration of denim textile wastewater for reuse. J. Hazard. Mater..

[bib0091] Saritha V., Maruthit Y.A., Mukkanti K. (2010). Potential fungi for bioremediation of industrial effluents. Bioresources.

[bib0092] Sasi P.K.C., AmbilyViswanathan J.M., Thomas D.M., Jacob J.P., Paulose S.V. (2020). Phycoremediation of paper and pulp mill effluent using planktochlorellanurekis and chlamydomonasreinhardtii–a comparative study. J. Environ. Treat. Tech..

[bib0093] Savant D.V., Abdul-Rahman R., Ranade D.R. (2006). Anaerobic degradation of adsorbable organic halides (AOX) from pulp and paper industry wastewater. Bioresour. Technol..

[bib0094] Scholes E., Carter P.B., Verheyen T.V. (2019). Colloidal carbon interference in the treatability of pulp and paper wastewater by MBR. J. Environ. Chem. Eng..

[bib0095] Sell N.J., Norman J.C., VandenBusch M.B. (1994). Removing color and chlorinated organics from pulp mill bleach plant effluents by use of flyash. Resour. Conserv. Recycl..

[bib0096] Shankar S., Ratnakar A., Singh S., Rawat S. (2020). Bioremediation of Industrial Waste For Environmental Safety.

[bib0097] Singh G., Arya S.K. (2019). Utility of laccase in pulp and paper industry: a progressive step towards the green technology. Int. J. Biol. Macromol..

[bib0098] Singh S. (2018). Fungal Biorefineries.

[bib0099] Singhal A., Thakur I.S. (2009). Decolourization and detoxification of pulp and paper mill effluent by cryptococcus sp. Biochem. Eng. J..

[bib0100] Tarlan E., Dilek F.B., Yetis U. (2002). Effectiveness of algae in the treatment of a wood-based pulp and paper industry wastewater. Bioresour. Technol..

[bib0101] Tarlan E., Yetis Ü., Dilek F.B. (2002). Algal treatment of pulp and paper industry wastewaters in SBR systems. Water Sci. Technol..

[bib0102] Thompson G., Swain J., Kay M., Forster C.F. (2001). The treatment of pulp and paper mill effluent: a review. Bioresour. Technol..

[bib0103] Tiku D.K., Kumar A., Chaturvedi R., Makhijani S.D., Manoharan A., Kumar R. (2010). Holistic bioremediation of pulp mill effluents using autochthonous bacteria. Int. Biodeterior. Biodegrad..

[bib0104] Toczyłowska-Mamińska R. (2017). Limits and perspectives of pulp and paper industry wastewater treatment–a review. Renew. Sustain. Energy Rev..

[bib0105] Torrades F., Peral J., Perez M., Domenech X., Garcia Hortal J.A. (2001). Removal of organic contaminants in bleached kraft effluents using heterogeneous photocatalysis and ozone. TAPPI J. (USA).

[bib0106] Tyagi S., Kumar V., Singh J., Teotia P., Bisht S., Sharma S. (2014). Bioremediation of pulp and paper mill effluent by dominant aboriginal microbes and their consortium. Int. J. Environ. Res..

[bib0107] Usha M.T., Chandra T.S., Sarada R., Chauhan V.S. (2016). Removal of nutrients and organic pollution load from pulp and paper mill effluent by microalgae in outdoor open pond. Bioresour. Technol..

[bib0108] Vijayakumar V., Balasubramanian N. (2015). Heavy metal removal by electrocoagulation integrated membrane bioreactor. Clean.

[bib0110] Virkutyte J. (2017). Current Developments in Biotechnology and Bioengineering.

[bib0111] Yadav S., Chandra R. (2015). Syntrophic co-culture of Bacillus subtilis and klebsiella pneumonia for degradation of kraft lignin discharged from rayon grade pulp industry. J. Environ. Sci..

[bib0112] Yao W.X., Kennedy K.J., Tam C.M., Hazlett J.D. (1994). Pre-treatment of kraft pulp bleach plant effluent by selected ultrafiltration membranes. Can. J. Chem. Eng..

[bib0113] Zainith S., Purchase D., Saratale G.D., Ferreira L.F.R., Bilal M., Bharagava R.N. (2019). Isolation and characterization of lignin-degrading bacterium Bacillus aryabhattai from pulp and paper mill wastewater and evaluation of its lignin-degrading potential. 3 Biotech.

